# Ionic Diffusiophoresis of Active Colloids via Galvanic Exchange Reactions

**DOI:** 10.1021/acs.nanolett.5c01567

**Published:** 2025-04-29

**Authors:** Zuyao Xiao, Juliane Simmchen, Ignacio Pagonabarraga, Marco De Corato

**Affiliations:** † Freigeist Group, Physical Chemistry, Technische Universität Dresden, Dresden 01069, Germany; ‡ Pure and Applied Chemistry, University of Strathclyde, Glasgow G11XL, U.K.; ¶ Departament de Física de la Matèria Condensada, Universitat de Barcelona, Barcelona 08028, Spain; § Aragon Institute of Engineering Research, University of Zaragoza, Zaragoza 50018, Spain; ∥ Universitat de Barcelona Institute of Complex Systems, Universitat de Barcelona, Barcelona 08028, Spain

**Keywords:** active matter, active colloids, self-propelled particles, ionic diffusiophoresis, ion transport, galvanic replacement

## Abstract

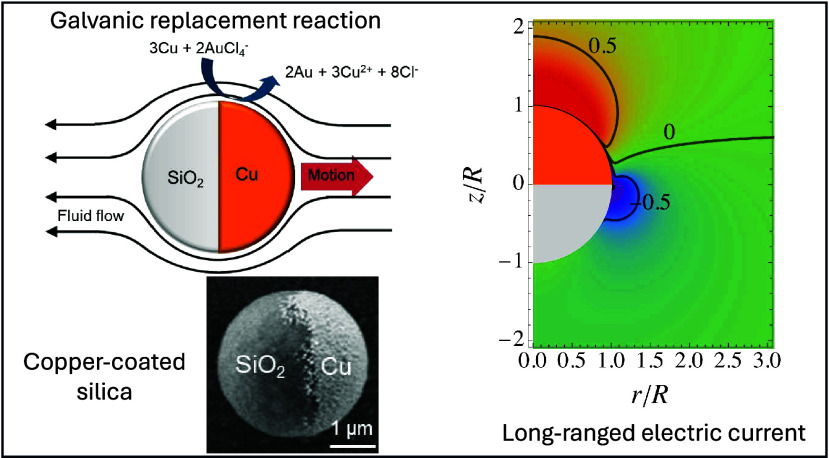

In order to move toward realistic applications by extending active matter propulsion reactions beyond the classical catalytic hydrogen peroxide decomposition, we investigate the self-propulsion mechanism of Janus particles. To address the influences of ionic species, we investigate Janus particles driven by a galvanic exchange reaction that consumes and produces ions on one hemisphere. Our galvanophoretic experiments in the regime of thin Debye layers confirm that even the simplest models in active matter are still full of important surprises. We find a logarithmic speed dependence on the fuel concentration, which cannot be explained using the classic ionic self-diffusiophoretic framework. Instead, an approach based on the Poisson–Nernst–Planck equations yields a better agreement with the experiments. We attribute the discrepancy between the two models to the breakdown of two key hypotheses of the ionic self-diffusiophoretic approach.

Traditionally, chemically active colloids propel autonomously through fluids by harnessing energy from a catalytic hydrogen peroxide (H_2_O_2_) degradation on one hemisphere surface.^1,2^ These artificial colloids have been envisioned for a range of applications from biomedical^3−5^ to environmental remediation,^6−8^ which is strongly limited by the use of strongly oxidizing H_2_O_2_ as fuel. Efforts to broaden the available chemical reactions range now from polymerization reactions^9^ to photodeposition of metals^10^ and enzymatic reactions.^11^ The implications of these different chemistries are only beginning to be understood.

In almost all of these instances, the active colloids propel in water driven by surface chemical reactions that consume and generate ionic species. These experimental results have so far been rationalized using the classic ionic diffusiophoretic framework.^12−18^ This approach assumes that the motion of the active colloid is driven by the interactions between the ionic species and the particle surface within a thin double layer.^19^ Nevertheless, despite the extensive modeling efforts, many questions on the mechanism responsible for their motion remain open.^20^ Tackling these open questions is crucial to ensure their successful technological applications.

The original self-diffusiophoretic framework was derived to describe the motion of colloids under external gradients of ionic species.^21^ To what extent the same theory applies to colloids that generate their own gradient via a chemical reaction remains an open question. This is particularly relevant for the case of ionic species involved in the reaction because their transport couples to the electric field. Such coupling effectively introduces correlations between ions,^22,23^ which can lead to self-propulsion even when the colloid is not charged.^18,24^

Here, we shed light on the mechanism propelling chemically active particles that consume and produce ionic species. We perform experiments using galvanophoretic Janus particles^25^ as a model system that creates flows based on ion release from a surface reaction. We rationalize the experimental results by comparing an ionic self-diffusiophoretic model with a more general Poisson–Nernst–Planck (PNP) model, which is computationally more expensive.

In the experiment, a solution of 3-μm-diameter copper-coated silica (Cu@SiO_2_) Janus particles (Figure 1a) and chloroauric acid (HAuCl_4_) are sequentially added to a glass substrate, and the motion of the particles is observed under a microscope; see details in the Supporting Information (SI). In the presence of HAuCl_4_, the Cu layer of the Janus particle undergoes galvanic replacement. This is a redox reaction in which one metal corrodes by losing electrons (oxidation) upon contact with the ions of another metal in solution. This process involves two key components: the first metal acts as an anode, while the ions of the second metal gain electrons (reduction) and are deposited onto the cathode. The reaction is driven by the difference in reduction potentials of the two metals; the second metal must have a higher reduction potential than the first one.

**Figure 1:**
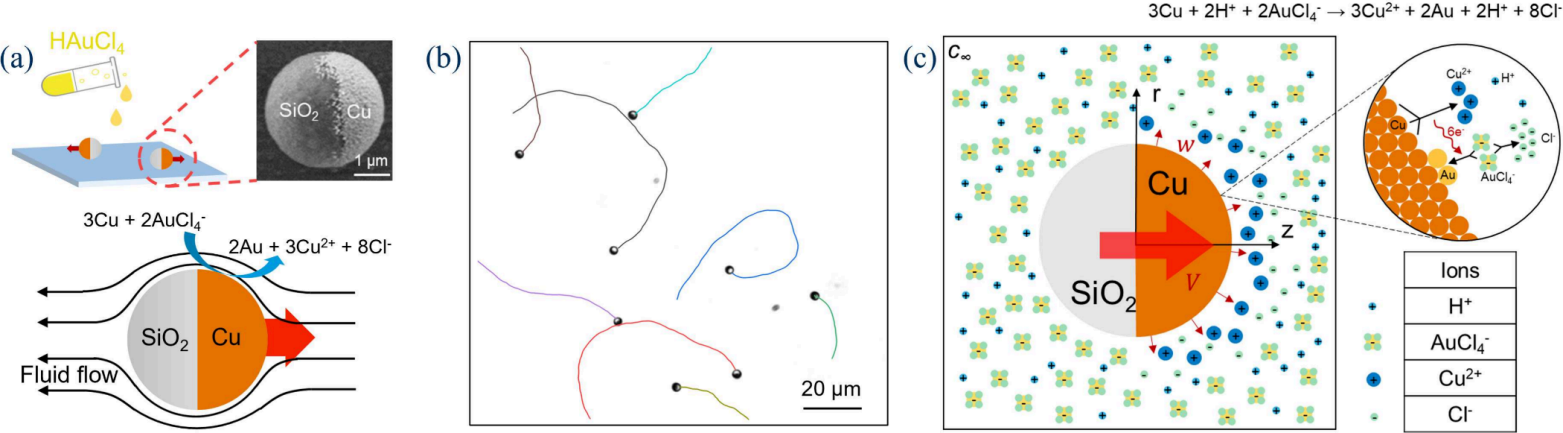
(a) Principle of the galvanophoretic experiment and the schematic flow field, with an SEM micrograph of a Cu@SiO_2_ Janus particle. (b) Exemplary trajectories of Cu@SiO_2_ Janus particles moving in 0.01 mM HAuCl_4_. (c) Schematic description of the chemical reaction and the features of the modeling approach. The inset highlights the galvanic replacement on the Cu surface of the active particle.

Here, Cu, with a reduction potential of 0.34 V vs the standard hydrogen electrode (SHE), acts as the anode. Au, with a reduction potential of 1.50 V vs SHE, acts as the cathode. Consequently, Cu is oxidized to Cu^2+^ ions, which are released into the solution, while Au is reduced to Au atoms and deposited onto the Cu surface, concurrently releasing Cl^–^ ions. As Cu^2+^ and Cl^–^ ions continue to be released and with the presence of excess HAuCl_4_, additional ions such as H^+^ and AuCl_4_^–^ are introduced into the solution. The overall galvanic replacement reaction scheme is described by the reaction^25,26^
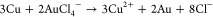
1occurring on the Cu side of the Janus particle. As a result of the galvanic replacement reaction occurring only on half of the surface, the Janus particle propels. Figure 1b shows typical trajectories of Janus particles moving in HAuCl_4_. The flow leading to particle motion most likely involves interactions between charged species produced and consumed at the Cu hemisphere and the particle surface, as well as the different diffusion coefficients of the products, causing an electric field.^25^

Previous studies on catalytic systems showed that increasing the ionic strength of the fluid generally decreases the speed of the active particle.^14,16,24,27−30^ Here, the electrolyte that sets the ionic strength of the solution is also the fuel that causes the propulsion. However, this is counteracted by a decrease of the screening length of electrostatic interactions, which weakens them.

As shown in Figure 2, by varying HAuCl_4_ concentrations from 10^–3^ to 1 mM in deionized water, we observed an increase in the particle velocity with rising HAuCl_4_ concentration. The dependency of particle speed on fuel concentration appears linear on a lin-log scale over almost 3 orders of magnitude, indicating a logarithmic speed dependence on *c*_∞_. This is in stark contrast to the case of particles propelling by autoelectrophoresis^19,27^ and by neutral diffusiophoresis,^31−34^ whose speed depends linearly on the fuel concentration. At large fuel concentrations, the particles are closer to the bottom wall because the electrostatic repulsion becomes increasingly screened. The reduction of the gap likely leads to an increase in friction from the bottom wall and a reduction of speed. Indeed, we found that some particles irreversibly stick to the bottom wall for concentrations of fuel larger than 0.1 mM, with their number increasing with fuel concentration.

**Figure 2:**
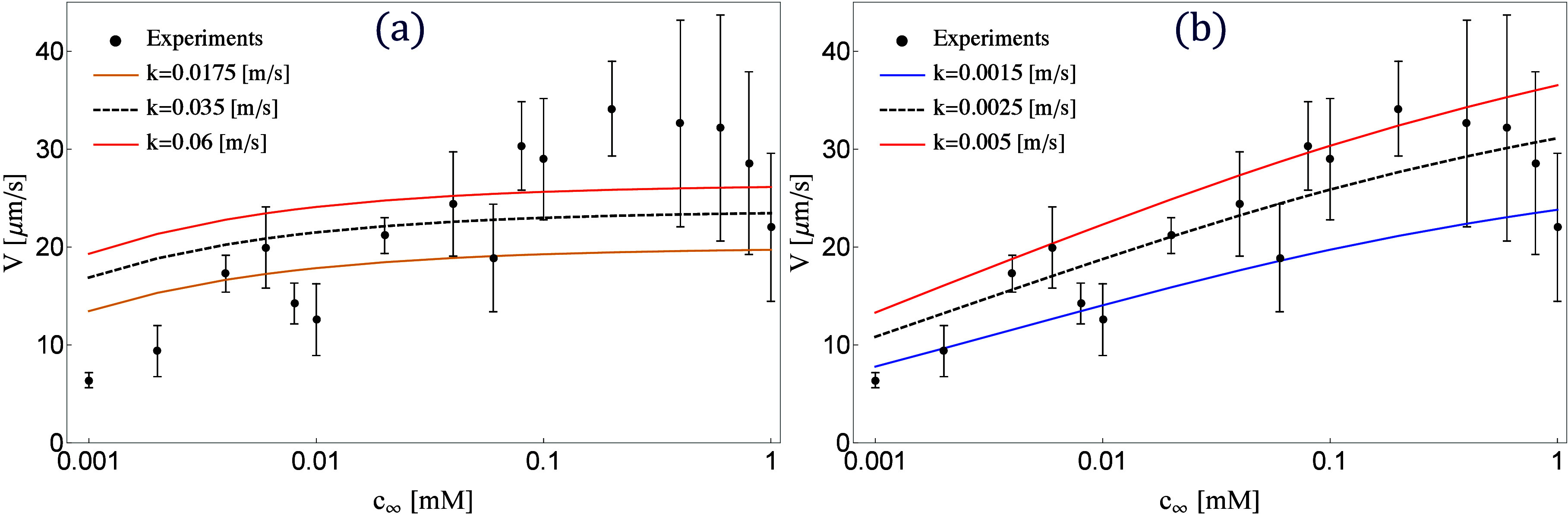
Comparison between the self-propulsion speed measured in the experiments and that obtained using two different theoretical models. (a) Velocity predicted by the self-diffusiophoretic model. (b) Velocity predicted by the PNP model. The black dashed lines represent the predictions of the best fit of *k*.

To better understand the origin of the logarithmic increase of the speed as a function of the fuel concentration, we employ modeling and simulations. We consider a Janus spherical colloidal particle of radius *R* with surface ζ potential Φ_s_ on the Cu side and a constant charge density, *q*_s_, on the silica side (Figure 1c). The active particle is suspended in an electrolyte solution given by the fuel. The electrolyte used in the experiments, HAuCl_4_, is a salt with unit valence and number density *c*_∞_, which is dissolved in a polar solvent with shear viscosity η. Given that HAuCl_4_ is a strong acid, we assume that the electrolyte fuel is completely dissociated into H^+^ and AuCl_4_^–^. We choose a cylindrical coordinate system with the origin at the particle center and the *z* axis representing the axis of cylindrical symmetry (Figure 1c).

The reaction at the Cu side of the Janus particle consumes two molecules of AuCl_4_^–^ and generates three Cu^2+^ ions and eight Cl^–^ ions. At the same time, the Cu coating is slowly replaced by Au.^25^ We characterize the ionic species by their concentration *c*_*i*_ with *i* = H^+^, Cl^–^, Cu^2+^, and AuCl_4_^–^. Following previous studies,^35^ we assume that the galvanic replacement is a first-order reaction with rate *w* = *kc*_AuCl_4_^–^_, where *k* is the reaction kinetic coefficient. We neglect the advective transport of ions because the characteristic Péclet number, *Pe* = *VR*/*D* ≈ 10^–2^, is much smaller than 1. In our estimate, we used a characteristic ionic diffusion coefficient *D* ≈ 10^–9^ m^2^/s and the maximum experimental velocity *V* ≈ 50 μm/s.

After the thickness of the double layer is estimated to be around λ_D_ ≈ 300–10 nm (Figure S1), it appears justified to employ the thin-layer approximation developed by Anderson and Prieve^12^ for 1:1 electrolytes and then extend it to a solution of electrolytes of arbitrary valence.^36,37^ This ionic self-diffusiophoretic approach has been widely used to analyze the self-propulsion of active particles.^13−19^

Following the ionic self-diffusiophoretic approach, the fluid domain is split into a charged inner layer near the surface of the particle and an outer layer representing the fluid bulk. In the outer layer, the fluid is neutral and the electric current, *I*, is locally zero: *I* = *e*∑_*i*_*z*_*i*_*J*_*i*_ = 0, with *J*_*i*_ the flux of the ionic species *i*. One can solve for their concentration in the outer layer (see the SI). The concentration in the inner layer is given by the Boltzmann distribution that matches the appropriate value at the outer layer. Because the ionic species have different diffusivities and valences, an electric field develops in the bulk to enforce electroneutrality.^12^ The electric field and the ion concentration gradient parallel to the particle surface drive an apparent slip velocity, which, in turn, drives the motion of the active particle due to momentum conservation.^21^ In our analysis, we included the contribution to the particle velocity originating from the outer layer discovered by Asmolov et al.^18^ Nevertheless, this term has a minimal contribution to the particle velocity, as observed by the authors.^18^ The full equations governing this model and the numerical method used to solve them are reported in the SI. All parameters are measured experimentally or are known and listed in Table S1. The only free parameter in this model is the kinetic constant *k* of the surface reaction.

In Figure 2a, we report with a dashed line the velocity of the active particle predicted by the ionic self-diffusiophoretic for *k* = 0.035 m/s, which gave the best fit using a least-squares method. We also plot the speed predicted by values of *k* that are larger and smaller than *k* = 0.035 m/s. The coefficient of determination of the least-squares minimization is , which means that this model fits the experimental data only marginally better than a horizontal line. The Damköhler number, *Da* = *kR*/*D*_HAuCl_4__, associated with the value of the kinetic constants displayed in Figure 2a, is between 17 and 60. These values correspond to cases where the chemical reaction is significantly faster than the diffusion of the species. The model predicts a weaker dependence of the particle propulsion speed on the fuel concentration in the far field, *c*_∞_, than that observed in the experiments. Because the double layer thickness, λ_D_, decreases as the fuel concentration, *c*_∞_, is increased (Figure S1), one would expect the ionic self-diffusiophoretic model to become a better description of the experimental system at large *c*_∞_. Instead, for large values of *c*_∞_, the simulations predict a constant velocity, independent of *c*_∞_. This discrepancy cannot be remediated by changing the main simulation parameters. Numerical simulations performed by changing the diffusion coefficients also predict a constant particle speed at large fuel concentrations (Figure S6).

To better understand the discrepancy between the ionic diffusiophoretic model and the experiments, we go beyond the thin-layer approximation and solve the full PNP equations. This approach keeps the same physical ingredients but lifts the key assumptions that the charges are confined to a thin layer and that the ionic charge distribution is the equilibrium one, described by the corresponding Boltzmann weight. Using the PNP approach, one solves for the concentration and the electrostatic potential around the active colloid with the boundary conditions applied at the surface of the colloid. The full set of equations and the numerical method used to solve it are given in the SI. The disadvantage of the PNP approach is that highly refined meshes are needed to capture the steep gradients near the particle surface (Figure S3).

In Figure 2b, we report the comparison between the velocity predicted by the full PNP equations and the experimental measurements for different values of the reaction kinetic constant, with the best fit given by *k* = 0.0025 m/s. We expected the PNP model to match the experiments as poorly as the ionic self-diffusiophoretic model because the two approaches should be equivalent when the thickness of the double layer is small. Instead, the numerical simulations using the PNP model quantitatively predict the growth of the particle velocity and display a logarithmic scaling over most of the fuel concentrations investigated in the experiments. At the largest fuel concentrations, the variability of the particle speed is larger and the agreement between experiments and simulations is only qualitative. For large values of *c*_∞_, the concentration of ionic species and their gradients near the reactive Cu side become large. The finite size of the ions and ion–ion correlation, which are ignored in the PNP approach, might become relevant. We also ignored the potential drop within the Stern layer at the reactive surface.^27,28,38^ Nevertheless, the comparison between parts a and b of Figure 2 clearly shows that the PNP description of ionic self-propulsion matches better the experiments than the predictions of the ionic self-diffusiophoresis.

In what follows we investigate why the PNP model fits better the experiments than the self-diffusiophoretic model. One possibility is that some of the assumptions made in the ionic self-diffusiophoretic model break down. In Figure 3a, we show the charge density, ρ = *e*∑_*i*_*z*_*i*_*c*_*i*_, for the case of *c*_∞_ = 0.04 mM and *k* = 0.003 m/s obtained from the simulation of the PNP model. For this value of the parameters, the Damköhler number *Da* = 3.21 and the thickness of the Debye layer is λ_D_ = 40 nm, which is almost 40 times smaller than the particle radius. We find that the charge density is confined within a very thin layer near the Cu side of the surface while the rest of the fluid is neutral. This result confirms that the fluid divides into a thin charged fluid layer near the particle surface and a neutral bulk, as assumed in the ionic self-diffusiophoretic framework.

**Figure 3:**
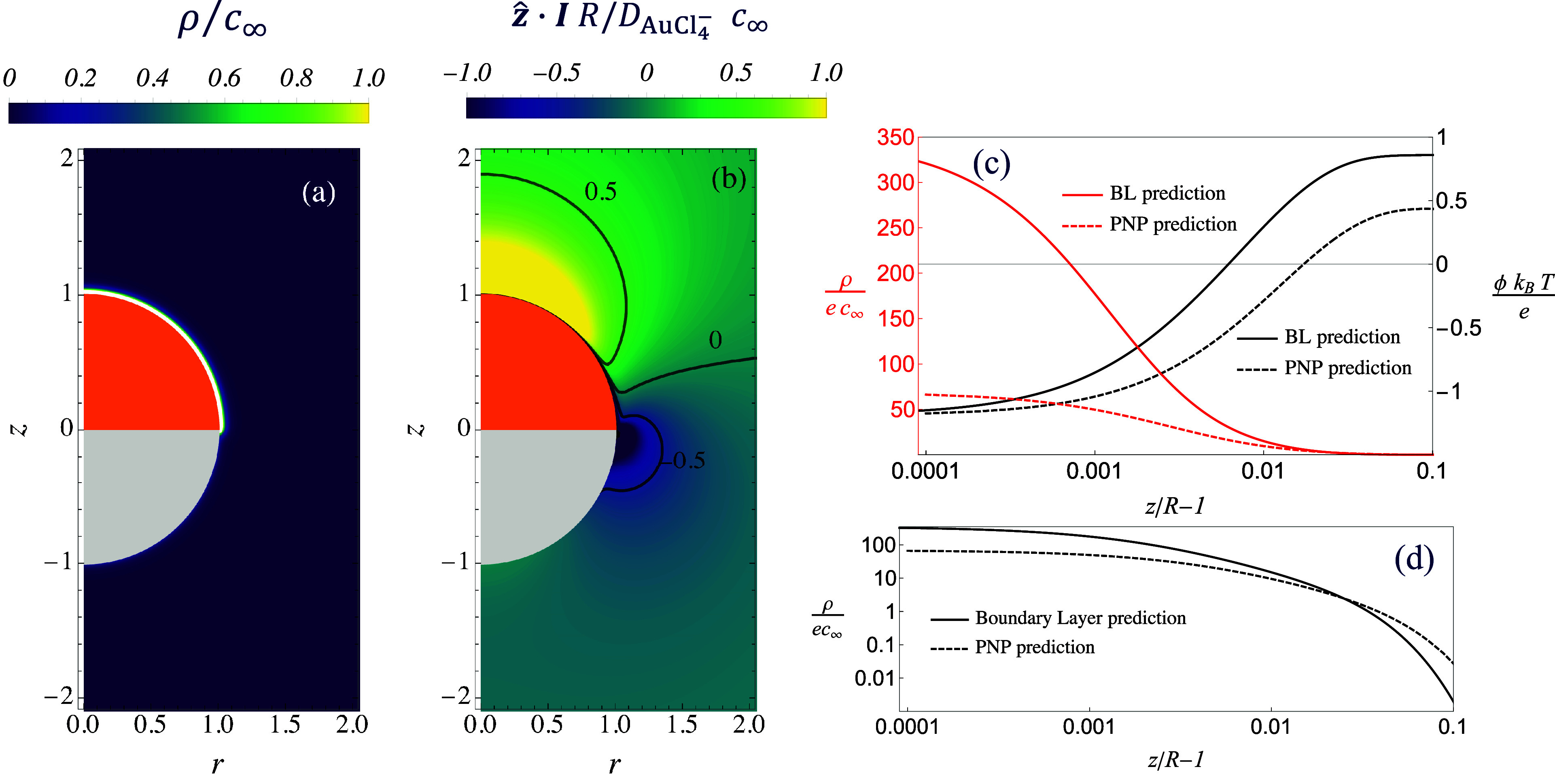
(a) Dimensionless charge density, ρ = *e*∑_*i*_*z*_*i*_*c*_*i*_, around the active colloid obtained from the solution of the PNP model. (b) *z*-component of the electric current obtained from the solution of the PNP equations. (c) Dimensionless charge density, ρ, and electrostatic potential, ϕ, along the *z* axis in a region close to the particle surface. (d) Log–log plot of the charge density. In parts c and d, the solid curves represent the predictions by the ionic self-diffusiophoretic (BL), while the dashed curve represents the prediction of the PNP approach. The parameters used in these figures are *c*_∞_ = 0.04 mM and *k* = 0.003 m/s. In panels a and b, the yellow region represents the Cu cap and the white region corresponds to the SiO_2_ hemisphere of the Janus particle.

Next, we investigate the electric current in the bulk of the fluid. According to the ionic self-diffusiophoretic framework, the current should vanish outside the thin charged layer.^36,37^ To verify this hypothesis, in Figure 3b, we plot the *z* component of the dimensionless electric current, ***I***·***ẑ*** obtained through the PNP model. Surprisingly, we find that there is a local nonzero electric current that extends into the outer layer. The electric current integrates to zero over the entire domain, as expected since no net current flows in or out of the domain. Previous works showed that local electric current could arise in the case of nonparallel gradients of multiple ionic species.^39,40^ It would be interesting to further investigate possible connections between these previous studies and our findings. To demonstrate the contribution of the outer layer current to the propulsion, we conduct the control experiments by measuring particle velocities at various concentrations of HAuCl_4_ in an additional 10 mM KCl electrolyte (Figure S5). The results show a suppression of particle velocity compared to the case in pure water, suggesting that the added background electrolyte weakens the electric current in the outer layer.

Finally, we inspect the electrostatic potential and the charge density within the thin layer next to the particle surface. In Figure 3c, we plot the electrostatic potential and the charge density along the *z* axis, focusing on a region close to the particle surface for the same value of the parameters used in Figure 3a,b. The solid lines represent the predictions of the ionic self-diffusiophoretic framework within the inner layer obtained by solving the Poisson–Boltzmann equation (eq 3 of the SI), with boundary conditions given by the matching with the outer fields. The dashed curves in Figure 3c represent the predictions of the PNP approach. We expect the PNP and ionic self-diffusiophoresis to give identical results within this small layer. Instead, the two modeling approaches predict different electrostatic potential profiles and charge densities near the particle surface. This finding suggests that the concentration of ionic species within the charged layer departs from the Boltzmann distribution, which is a key assumption of the ionic self-diffusiophoretic approach. This is confirmed in Figure 3d, where the charge density is shown to decay slower with the distance than that predicted by the Boltzmann distribution.

We hypothesize that the breakdown of these two hypotheses is responsible for the different predictions of the two approaches shown in Figure 2. Indeed, the gradients of ionic species in the outer layer contribute to the driving force of the phoretic flows and the charge density inside the inner layer determines their magnitude. Consequently, a different prediction of these two quantities will lead to different flow fields and thus to different particle speeds.

In conclusion, we performed experiments with micron-sized Cu@SiO_2_ Janus particles, which undergo a galvanic exchange reaction in the presence of HAuCl_4_ that consumes and releases ionic species. The reaction on the Cu side leads to autonomous propulsion of the Janus particle at a speed that depends logarithmically on the fuel concentration over 3 orders of magnitude. We rationalized the experimental results using two theoretical models: one based on the classic ionic diffusiophoretic framework and one based on the PNP approach. Although the two modeling approaches are considered to be equivalent in the limit of thin double layers, the logarithmic particle speed trend cannot be explained by the classic ionic self-diffusiophoretic model. Only the model based on the PNP equations captures the main experimental observations. We trace the origin of this discrepancy between the two modeling approaches to two main differences. First, we find that the PNP approach predicts a local current in the bulk of the fluid, which is assumed to be zero in the ionic self-diffusiophoretic framework. Second, we find that the PNP and ionic self-diffusiophoretic approaches predict different electrostatic potential and charge density profiles in the region near the particle surface. This implies that the ion concentration in the charged layer does not follow the Boltzmann distribution, as assumed in the self-diffusiophoretic framework. The distinct profiles in the inner charged layer are responsible for the different particle speeds predicted by the two models. While the ionic diffusiophoretic approach has been successfully employed to model passive particles exposed to external gradients, our results imply that this approach breaks down in the case of reactive active particles driven significantly out of equilibrium in an ionic fuel.

## Supporting Information Available

Experimental methods and equations governing the models used in the main text (PDF)
