# Supplementary material for: Ionic Diffusiophoresis of Active Colloids via Galvanic Exchange Reactions
Source: Nano Lett. 2025 Apr 29;25(19):7975–80. doi: 10.1021/acs.nanolett.5c01567 (PMC12082695; doi:10.1021/acs.nanolett.5c01567)
Supplement: Supplementary file 1 — nl5c01567_si_001.pdf [file nl5c01567_si_001.pdf]

Supplementary information to "Ionic diffusiophoresis of active colloids via galvanic exchange reactions"

Zuyao Xiao,† Juliane Simmchen,*,† Ignacio Pagonabarraga,‡ and Marco De Corato*,§

†*Pure and applied chemistry, University of Strathclyde, Glasgow, UK*

‡*Departament de Física de la Matèria Condensada, Universitat de Barcelona, Barcelona, Spain*

¶*Universitat de Barcelona Institute of Complex Systems (UBICS), Universitat de Barcelona, 08028 Barcelona, Spain*

§*Aragon Institute of Engineering Research (I3A), University of Zaragoza, Zaragoza, Spain*

E-mail: juliane.simmchen@strath.ac.uk; mdecorato@unizar.es

Experimental methods

Janus Cu@SiO_2 particles were synthesized following the previous method[1,2] by thermal depositing a copper layer on half of each 3 µm diameter SiO_2 particle (Sigma No. 44054-5ML-F). Initially, SiO_2 particles were dispersed in ethanol and sonicated. Subsequently, the suspension was drop-casted onto a plasma-treated glass slide to form a monolayer. Then a 50 nm copper layer was deposited on the SiO_2 monolayer to create Janus Cu@SiO_2 particles, which are finally sonicated and redispersed in deionized water. To measure the zeta potentials of the two sides of the Janus particles, we separately measured the zeta potentials of

pure Cu particles and pure SiO_2 particles using a Malvern Zetasizer in de-ionized water. The measurements yielded values of -35 mV for Cu and -40 mV for SiO_2, respectively. Converting -40 mV for SiO_2 to a charge density, we obtain a surface charge density $q_s = -30\mu C/\mathrm{m}^2$. The SEM image of Cu@$SiO_2$ particles indicates a smooth copper cap distinctly contrasting with the SiO_2 substrate. Also, the differential optical properties between Cu and SiO_2 result in the Cu appearing dark and SiO_2 appearing bright under microscopic examination.

Motion Experiments

For each experiment, 8 µL of Cu@SiO_2 suspension was pipetted onto the plasma-cleaned glass slide. Then the particles were allowed to sediment near the slide substrate, reaching a relatively stable height over time. Subsequently, 2 µL of $HAuCl_4$ solution at varying concentrations was rapidly added. The motion of particles was observed using an inverted microscope(Zeiss Axiobserver) and recorded using a camera (Zeiss Axiocam 702 Mono) at a frame rate of 30 frames per second.

Motion Analysis

The motion videos were processed using ImageJ and MATLAB to extract the x-y coordinates of each particle across sequential frames. The instantaneous speeds of the particles were then calculated as follows $v = \sqrt{(x_{i+1} - x_i)^2 + (y_{i+1} - y_i)^2}/\delta t$. Where x_i and y_i are the x and y coordinates of the particle at the previous frame, while x_{i+1} and y_{i+1} are the x and y coordinates of the particle at the next frame, δt is the time of one frame. The average speeds for each concentration were then calculated by averaging the instantaneous speeds for the first 150 frames over 10 particles.

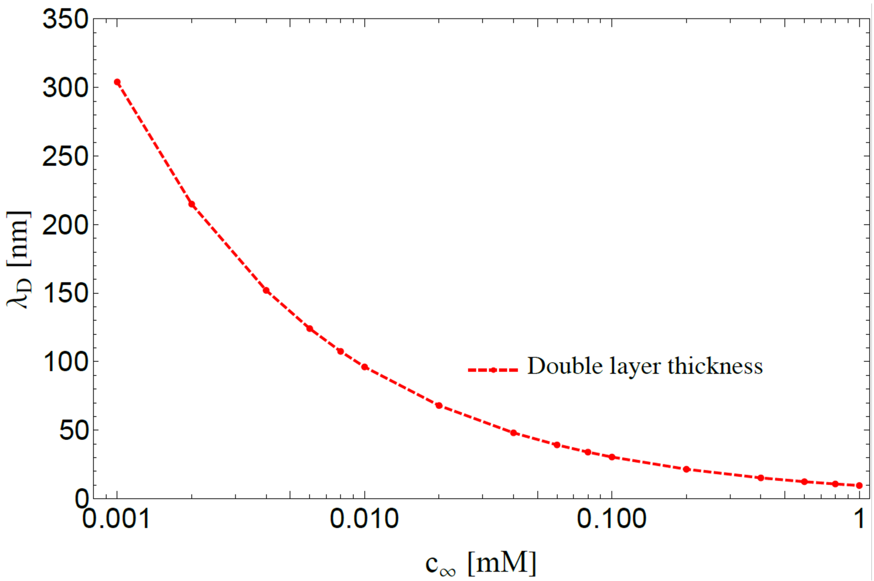

Figure S1: Estimate of the thickness of the double layer as a function of the fuel concentration in the solution.

Ionic self-diffusiophoretic model

In the self-ionic-diffusiophoretic approximation,we divide the fluid domain into a charged inner region and a neutral bulk. We define the concentration of the ionic species and the electrostatic potential in the inner region using lowercase variables and their values in the bulk using uppercase variables. Under this approximation, the concentration of all the ionic species in the inner boundary layer is given by the Boltzmann distribution that matches the outer concentration far from the particle surface[3]

$$c_i = C_i \exp\left[-z_i e\left(\phi - \Phi\right)/k_B T\right] \tag{1}$$

where ϕ is the electrostatic potential in the inner layer, c_i is the concentration of the ionic species in the inner layer and C_i is the concentration in the bulk region evaluated at $r = R^+$. In the inner layer a stretched coordinate, y, that goes from zero to infinity is used.[3] The limiting values of all the variables as $y \to \infty$ must match the value of the same variables in

the outer layer evaluated at $r = R^+$. In the inner layer the electrostatic potential, ϕ, satisfies

$$\epsilon d^2\phi/dy^2 = -\sum_i e\, z_i\, c_i \ , \tag{2}$$

with the sum running over all the ionic species i involved in the reaction with z_i their valence and the expression for c_i given by Eq. (1). The boundary condition on the copper side is given by the jump between the electrostatic potential in the outer layer and the surface potential of the particle $\phi(0) = \phi_s - \Phi$ at $y = 0$, where Φ is the electrostatic potential in the outer layer evaluated at $r = R^+$. The boundary condition on the silica side is more complicated because the surface charge density, q_s, is fixed rather than the surface potential. A relation between the surface potential and the surface charge is known in the case of Z:Z electrolytes but we are not aware of any relations in the case of an arbitrary solution of electrolytes. However, we note that the surface charge of the silica $q_s = -30\mu C/\mathrm{m}^2$ is small. This can be quantified by the dimensionless number $q_s/\sqrt{(2\epsilon k_B T c_\infty N_A)} \approx -0.16$ evaluated at the smallest value of c_∞ used in the experiments. As a consequence, we use the Debye-Huckel approximation to link the surface charge and the surface potential on the copper side $\phi_s = q_s\sqrt{(\epsilon k_B T)}/\sqrt{(e^2\, N_A\, \sum_i z_i^2 C_i)}$.

Following,[4] the apparent slip velocity developed at the outer edge of the inner layer is given by

$$\boldsymbol{v}_s = -\frac{\epsilon\, \phi(0)^2}{8\,\eta}\frac{\sum_i z_i^2 \nabla_\parallel C_i}{\sum_i z_i^2\, C_i} - \frac{\epsilon\, k_B T\, \phi(0)}{e\eta}\frac{\sum_i z_i\, D_i\, \nabla_\parallel C_i}{\sum_i z_i^2\, D_i\, C_i} \ , \tag{3}$$

where $\nabla_\parallel$ represents the gradient tangent to the particle surface. This equation is valid up to the second order in the jump of the electrostatic potential $\phi(0)$.[4] To compute the slip velocity one only needs to know the concentration of the ionic species C_i and the electrostatic potential Φ in the outer layer.

In the outer layer, the fluid is electroneutral so that the condition $\sum_i z_i C_i = 0$ must be satisfied locally. It follows that one can solve for all of the ionic species involved in the reaction minus one, and use the electroneutrality constraint to compute the remaining one.

At steady state, the ionic species satisfy drift-diffusion equations

$$D_i \nabla^2 C_i + \frac{z_i D_i e}{k_B T} \nabla \cdot C_i \nabla \Phi = 0 \tag{4}$$

The domain of validity of the equation is $r \in [R^+ \, \infty[$. The surface reaction is applied as a boundary condition at $r = R^+$ on the copper side

$$-\left(D_i \nabla C_i + \frac{z_i D_i e}{k_B T} C_i \nabla \Phi \right) \cdot \boldsymbol{n} = m_i \, w \ , \tag{5}$$

where m_i is the stoichiometric coefficient of the reaction, w is the reaction rate $w = k \, e^{\phi(0)} \, C_{\mathrm{AuCl}_4^-}$ and $\boldsymbol{n}$ is the vector normal to the particle surface. On the silica side of the active particle the boundary condition is given by

$$-\left(D_i \nabla C_i + \frac{z_i D_i e}{k_B T} C_i \nabla \Phi \right) \cdot \boldsymbol{n} = 0 \ . \tag{6}$$

Far from the active particle, $r \to \infty$, the concentration of the fuel is fixed $C_{\mathrm{AuCl}_4^-} = C_{\mathrm{H}^+} = c_\infty$ and the concentration of the products is zero $C_{\mathrm{Cu}^{2+}} = C_{\mathrm{Cl}^-} = 0$. We need an equation for the electrostatic potential, which can be obtained from the electroneutral constraint $\sum_i z_i C_i = 0$, giving the following equation for Φ

$$-\nabla \cdot \left(\frac{\sum_i z_i^2 C_i D_i e}{k_B T} \nabla \Phi \right) = \sum_i z_i D_i \nabla^2 C_i \ , \tag{7}$$

At infinity, $r \to \infty$, the electrostatic potential is zero $\Phi = 0$. At $r = R^+$ the conservation of charges in the reaction ensures that

$$-\left(\frac{\sum_i z_i^2 C_i D_i e}{k_B T} \nabla \Phi + \sum_i z_i D_i \nabla C_i \right) \cdot \boldsymbol{n} = 0 \ . \tag{8}$$

This set of equations ensures that the outer region is electroneutral and that the local electric current is zero $\sum_i z_i \boldsymbol{J}_i = \boldsymbol{0}$. The details of the numerical method employed are reported in

SI.

Once all of the outer fields are computed, we calculate the velocity of the active particle using the reciprocal theorem.[5] As pointed out in,[6] the particle velocity has a contribution due to the slip velocity and a contribution due to the charge density in the outer layer.

$$V = -\frac{1}{S}\int_S \boldsymbol{v}_s \cdot \boldsymbol{e}_z dS - \frac{\epsilon e}{6\pi\eta R}\int_\Omega \nabla^2\Phi\nabla\Phi\cdot(\hat{\boldsymbol{v}} - \boldsymbol{e}_z)d\Omega \tag{9}$$

where $\boldsymbol{e}_z$ is the unit vector in the z-direction, and $\hat{\boldsymbol{v}}$ is the velocity field generated by a sphere of the same radius as the active sphere moving along the $z-$axis with unit velocity. The parameters employed in the simulations are summarized in Table 1.

Table S1: Parameters used in the numerical simulations

Constant	Description	Value
$D_{\mathrm{AuCl}_4^-}$	Diffusion coefficient of $AuCl_4^-$	$1.4\ 10^{-9}\,\mathrm{m}^2/\mathrm{s}$
D_{H^+}	Diffusion coefficient of H^+	$9.23\ 10^{-9}\,\mathrm{m}^2/\mathrm{s}$
$D_{\mathrm{Cu}^{2+}}$	Diffusion coefficient of Cu^{2+}	$0.71\ 10^{-9}\,\mathrm{m}^2/\mathrm{s}$
D_{Cl^-}	Diffusion coefficient of Cl^-	$2.03\ 10^{-9}\,\mathrm{m}^2/\mathrm{s}$
Φ_s	Zeta potential of the Copper side	-35 mV
q_s	Surface charge density of the silica side	$q_s = -30\mu C/\mathrm{m}^2$
R	Radius of the active particle	1.5 μm
η	Viscosity of the liquid	10^{-3} Pa s
ϵ	Solution dielectric constant	$7.083\,10^{-10}$ F/m
T	Absolute temperature	300 K

Numerical details on the Ionic self-diffusiophoretic model

We solve this set of partial differential equations using the finite element method employing an axisymmetric computational domain between two concentric spheres and we use a cylindrical coordinate system. The axis of symmetry along which the particle can propel is the z-axis of the cylindrical coordinate system. To minimize the finite size effects, the far-field condition is applied to the outer sphere, which has a radius 500 times larger than the active particle. We discretize the domain using a body-fitted triangular mesh and we use quadratic interpolation for all of the variables. An example of the mesh used for the

calculations is shown in Figure S2 We use the Newton-Raphson method to solve the resulting nonlinear system of equations, starting from an initial guess $\Phi = 0$,$C_{\mathrm{AuCl}_4^-} = C_{\mathrm{H}^+} = c_\infty$, and $C_{\mathrm{Cu}^{2+}} = C_{\mathrm{Cl}^-} = 0$. We perform simulations at increasing values of the kinetic constant k and we use the previous solution as the initial guess of the Newton-Raphson iterations for the next simulation.

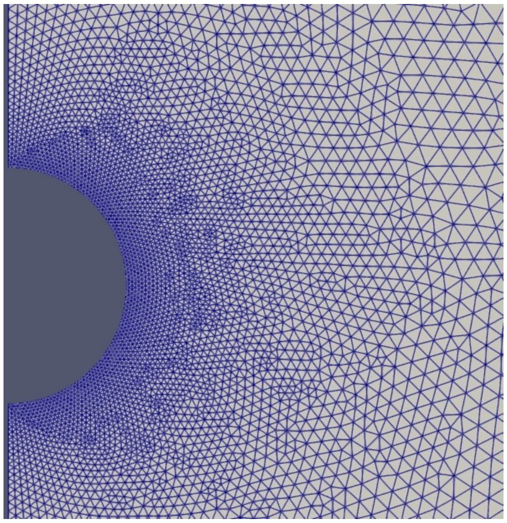

Figure S2: Example of the mesh used to solve the ionic self-diffusiophoretic model.

Poisson-Nernst-Planck (PNP) model

This approach does not make any assumption on the thickness of the charged layer near the particle surface and there is no need to define an inner and outer layer. As a consequence, all the variables are defined in the domain outside the particle $r \geq R$. The concentration of the ionic species, c_i, at steady state is determined by

$$D_i \nabla^2 c_i + \frac{z_i D_i e}{k_B T} \nabla \cdot c_i \nabla \Phi = 0 \ . \tag{10}$$

The electrostatic potential, ϕ, satisfies the Poisson's equation

$$\epsilon \nabla^2 \Phi = - \sum_i e \, z_i \, c_i \ , \tag{11}$$

with ϵ the permittivity of the liquid. On the copper side of the particle surface, $r = R$, the species react:

$$-\left(D_i \nabla c_i + \frac{z_i D_i e}{k_B T} c_i \nabla \Phi\right) \cdot \boldsymbol{n} = m_i \, w \ . \tag{12}$$

with $w = k \, c_{\mathrm{AuCl_4^-}}$ the reaction rate and m_i the stoichiometric coefficient. The silica side of the particle surface is impermeable to the ionic species

$$-\left(D_i \nabla c_i + \frac{z_i D_i e}{k_B T} c_i \nabla \Phi\right) \cdot \boldsymbol{n} = 0 \ . \tag{13}$$

The species concentration is specified far from the particle $r \to \infty$. The concentration of the fuel is fixed $c_{\mathrm{AuCl_4^-}} = c_{\mathrm{H^+}} = c_\infty$ and the concentration of the products is zero $c_{\mathrm{Cu^{2+}}} = c_{\mathrm{Cl^-}} = 0$. On $r = R$, we assume that the electrostatic potential is fixed $\phi = \phi_s$ on the copper side and the surface charge density is fixed on the silica side $\epsilon \nabla \phi \cdot \boldsymbol{n} = q_s$. This set of equations together with their boundary conditions gives a closed problem. The details of the numerical method employed are reported in SI. Once the solution is found, the velocity of the active particle is obtained from the generalized reciprocal theorem through the integral[5]

$$V = -\frac{\epsilon e}{6\pi\eta R} \int_\Omega \nabla^2 \Phi \nabla \Phi \cdot (\hat{\boldsymbol{v}} - \boldsymbol{e}_z) d\Omega \ . \tag{14}$$

Numerical details on the Poisson-Nernst-Planck (PNP) model

The governing equations are solved using the finite element method using an axisymmetric domain. The computational domain is given by the area between two concentric circles. The inner circle of radius R represents the particle surface. To minimize finite-size effects, the radius of the outer circle is chosen 1000 times larger than the particle radius. The computational domain is discretized into 29600 quadrilateral elements. To capture the steep variations of the variables in the liquid near the particle surface, we use a boundary layer

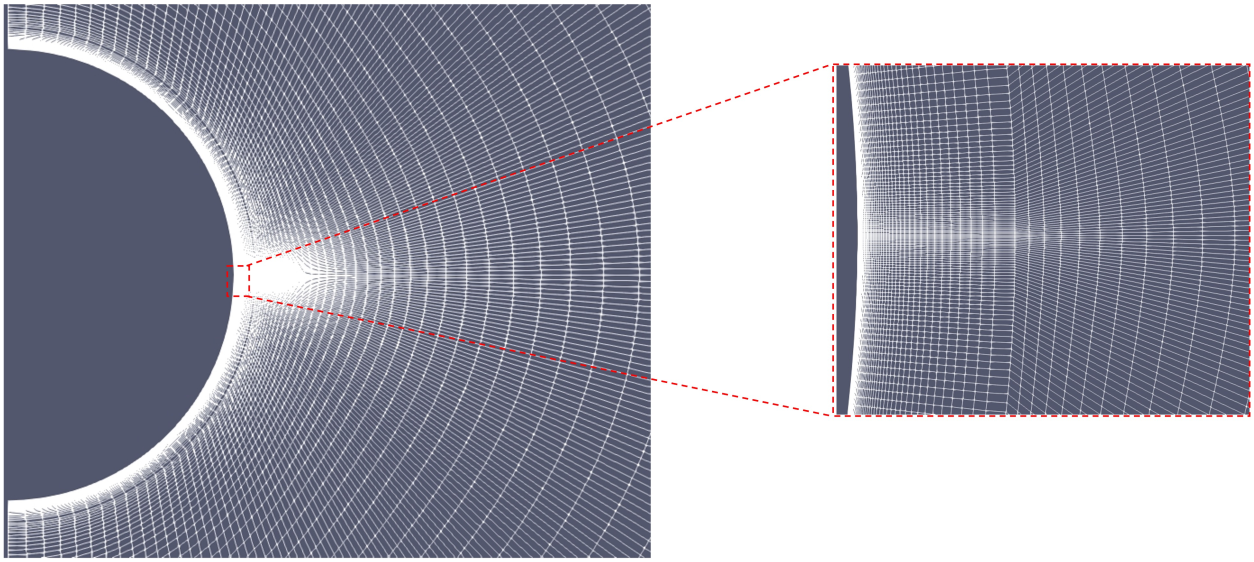

Figure S3: Example of the mesh employed to solve the equations governing the Poisson-Nernst-Planck model. To correctly capture the steep gradients, a very refined mesh is required near the particle surface. A refined mesh is also required near the equator of the Janus particle than at its poles.

approach whereby the element thickness in the radial direction is $10^{-4}R$. The thickness of the elements progressively increases far from the particle surface. An example of the mesh employed in the calculation is given in Figure S3. We use the Newton-Raphson method to solve the resulting nonlinear system of equations, starting from an initial guess $\phi = 0$,$c_{\mathrm{AuCl}_4^-} = c_{\mathrm{H}^+} = c_\infty$, and $c_{\mathrm{Cu}^{2+}} = c_{\mathrm{Cl}^-} = 0$. We perform simulations at increasing values of the kinetic constant k and we use the previous solution as the initial guess of the Newton-Raphson iterations for the next simulation. In Figure S4 we demonstrate that the mesh chosen yields convergent results. To find a convergent mesh, we find it critical to have a more refined mesh near the equator of the Janus particle where the surface of the particle transitions from copper to silica. In this region the surface reaction rate, zeta potential and surface charge change steeply along the azimuthal direction and a refined mesh is required to avoid numerical artifacts.

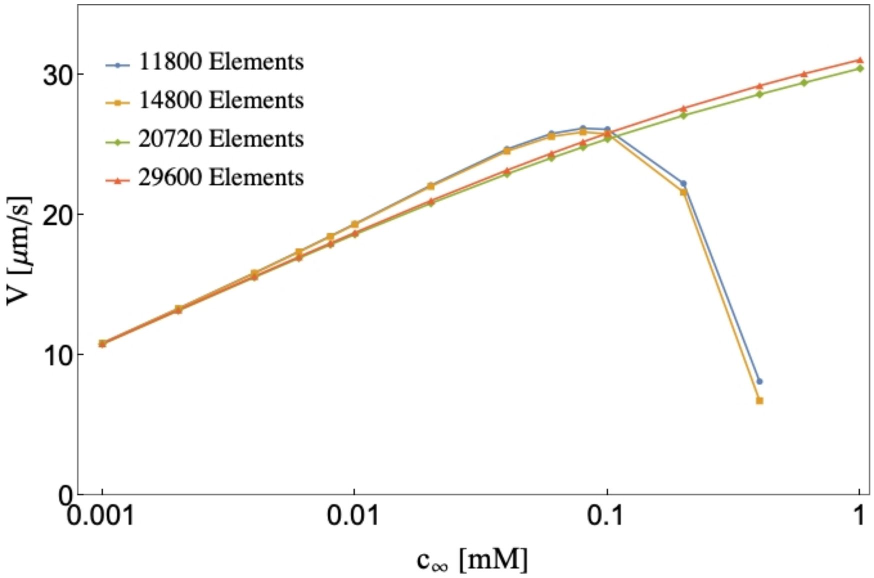

Figure S4: Mesh convergence for $k = 0.003$m/s.

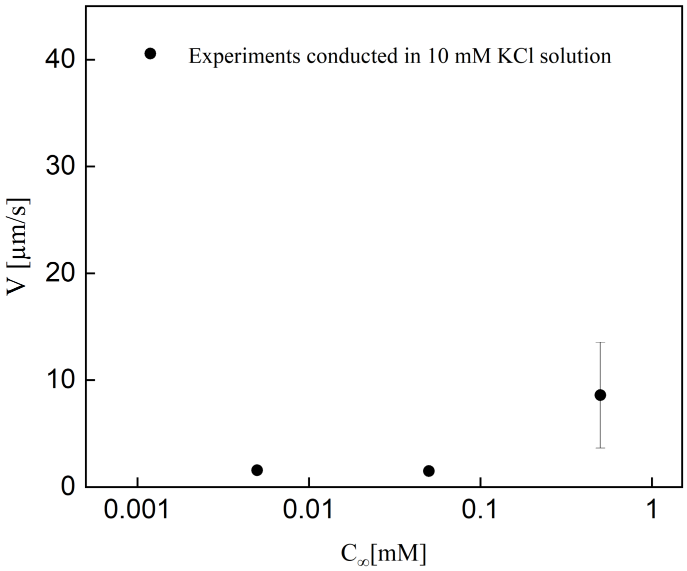

Figure S5: Particle velocity at various $HAuCl_4$ concentrations with 10 mM KCl as background electrolyte.

References

(1) Bastos-Arrieta, J.; Bauer, C.; Eychmüller, A.; Simmchen, J. Galvanic replacement induced electromotive force to propel Janus micromotors. *J. Chem. Phys.* **2019**, *150*.

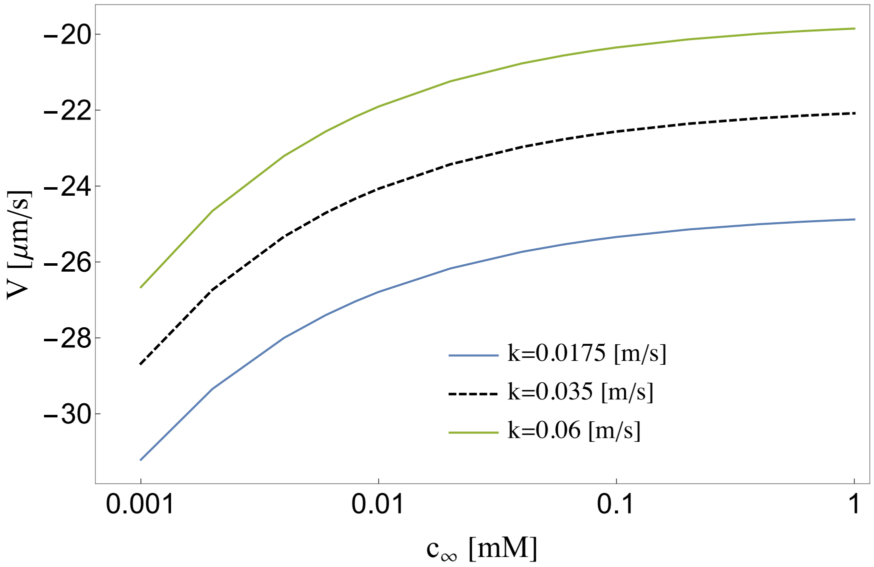

Figure S6: Particle velocity obtained through numerical simulations of the Ionic self-diffusiophoretic model considering different diffusion coefficient. In this figure, we artificially changed the H^+ diffusion coefficient to $D_{H^+} = 3.1\,10^{-9}$, the Cu^{2+} diffusion coefficient to $D_{Cu^{2+}} = 1.4\,10^{-9}$, and the Cl^- diffusion coefficient to $D_{Cl^-} = 1.015\,10^{-9}$. Changing the diffusion coefficient has changed the sign of the particle velocity but not the fact that its speed reaches an asymptote at large fuel concentrations.

(2) Feuerstein, L.; Biermann, C. G.; Xiao, Z.; Holm, C.; Simmchen, J. Highly efficient active colloids driven by galvanic exchange reactions. *J. Am. Chem. Soc.* **2021**, *143*, 17015–17022.

(3) Prieve, D.; Anderson, J.; Ebel, J.; Lowell, M. Motion of a particle generated by chemical gradients. Part 2. Electrolytes. *J. Fluid Mech.* **1984**, *148*, 247–269.

(4) Chiang, T.-Y.; Velegol, D. Multi-ion diffusiophoresis. *J. Coll. Int. Sci.* **2014**, *424*, 120–123.

(5) Masoud, H.; Stone, H. A. The reciprocal theorem in fluid dynamics and transport phenomena. *J. Fluid Mech.* **2019**, *879*, P1.

(6) Asmolov, E. S.; Nizkaya, T. V.; Vinogradova, O. I. Self-diffusiophoresis of Janus particles that release ions. *Phys. Fluids* **2022**, *34*, 032011.
